# Book and Pamphlet Notices

**Published:** 1858-09

**Authors:** 


### BOOK AND PAMPHLET NOTICES.

Bucknill and Tuke on Insanity. Pps. 530. Published by Blanchard & Lea, Philadelphia.

It would afford us great pleasure to present to the readers of the Journal some extracts and make a few comments on the above-named work, but we cannot devote the space necessary to do justice to the work. It is a systematic treatise on insanity, correctly and ably, yet concisely, representing all the improvements in the diagnosis, pathology and treatment of mental disease.

To give the reader an idea of its general scope and character, we enumerate and copy the heading of the different chapters.

Chapter I. gives an historical sketch of insanity among the nations of antiquity, mainly in regard to extent.

Chapter II. sums up the opinions of ancient medical men on the treatment of the insane.

Chapter III. considers modern civilization in its bearing upon insanity.

Chapter IV. is a picture of the difference in the management of the insane by the ancients and moderns, and shows the present ameliorated condition with reference particularly to mechanical restraints of this unfortunate class of afflicted.

Chapter V. speaks of the definition of insanity, and dwells upon the great difficulty of properly classifying different cases so as to systematize the subject. This is an excellent chapter, and freely and ably discusses the subject.

Chapter VI. enumerates the various forms of mental disease.

Chapter VII. is devoted to the statistics and causes of insanity, proportion of recoveries, proportion of cases to the population in different countries, mortality of the insane, etc.

The chapters enumerated above are the production of Dr. Tuke, while all those that follow were written by Dr. Bucknill. They consist of Chapters VIII. on Diagnosis, IX. Pathology, and X. Treatment of Insanity. They are readable, thoroughly practical and reliable.

The work is wound up by an appendix, containing cases illustrated by portraits in the frontispiece. These portraits show the expression of different forms of insanity in the face, and of course are valuable assistance so far as they go.

It also contains cases illustrative of the treatment and management recommended by the authors as modern representatives of the profession. Also, cases illustrating causation and pathology, so far as may be done without clinical observation.

This book, we think, is just such an one as the profession needs, and emanating from the source it does, have no hesitancy in recommending it to any of our readers who desire to be supplied with the information becoming indispensably necessary in regard to insanity. It is not intended particularly for the specialist in this department of medicine, although full and thorough, but is adapted more especially to the wants of the general practitioner. It may be bought of Mr. Keen, of this city. W. H. B.

A Practical Treatise on the Causes, Symptoms and Treatment of Spermatorrhea. By M. Lallemand, formerly Professor of Clinical Surgery at the University of Montpelier, etc. Translated by Henry J. McDougall, Member of the Royal College of Physicians of England, etc., etc. Third American Edition, to which is added, "On Diseases of the Vesiculæ Seminales and their Associated Organs," with special reference to the Morbid Secretions of the Prostatic and Urethral Mucous Membrane, by Morris Wilson, M. D. Philadelphia: Blanchard & Lea. 1858. Pps. 380.

This excellent book should be in the library of all who undertake the responsible position of medical adviser. With reference to the subject upon which it treats, it is *the book*. On account of the importance of the subject, and with the hope of inducing a close study of this distressing assemblage of diseases, we give below a summary of impressions received from reading the book and the limited experience we have enjoyed. We desire to premise that it is very imperfect, and will only serve—we hope—to excite our readers to a desire for a complete acquaintance with it.

The symptoms are general and local. The main and characteristic local symptoms are when the disease is fully developed—loss of venereal desires, imperfect secretions, imperfect seminal ejections, seminal sediment in the urine, seminal extrusion during defecation, unconscious seminal dribbling or discharge during lascivious dreams or thoughts at night, soreness or other abnormal sensations in the urethra or prostate gland or both. The early symptoms are the same, but more moderate in degree, the venereal erethism being rather uncommon, and leaving unpleasant sensations behind. When discharged in the urine, by letting this fluid stand, the semen will appear as a white granular sediment at the bottom. By gently decanting the urine, this sediment may be obtained in form sufficiently concentrated for microscopic examination. When discharged during defecation, it drops from the urethra in quantities varying from a few drops to a handful, and seems to be pressed out by the hardened fæces bearing strongly upon the distended and relaxed vesiculæ seminales. Or, when the discharge is effected on account of painful irritation of the rectum, it may be thrown off in jets. These are the common forms of diurnal emissions, and are regarded as the most destructive in their general effects of all others, and often overlooked or mistaken for something else. The nocturnal emissions are next most injurious, and are recognizable by the stains on the linen or the recollections of the patient. They may be accompanied by erection, and other energetic venereal manifestations, are involuntary and easier cured than the preceding variety. In regard to them it may be said, that more unconscious the patient is of them, the less venereal orgasm, the more injurious and obstinate they will prove. If attended with the vehemence and pleasurable sensations of ordinary venereal acts, they may be simply the effect of seminal redundance, and beneficial instead of injurious. When this is the case, the patient, instead of increased languor and uneasiness, will be in better spirits and more buoyant and healthful in feeling than before the occurrence. There is always also with these symptoms some soreness of the prostate testicles, urethra, or some other portions of the seminal mucous membrane.

The general symptoms are very various. Impotence is the most common, perhaps, in bad cases, either in consequence of inability for perfect coitus or unfit seminal fluid. The digestive organs in grave cases become profoundly affected, and the symptoms and nature of their diseases as diverse as possible.

Indigestion and constipation, with all their consequences, are particularly common. Nutrition and calorification deficient, the patient is emaciated, and subject to be affected with the slightest atmospheric changes. The respiratory and circulatory apparatus are so deranged as to simulate diseases of the lungs and heart, great muscular debility, derangement of sensation and power in the extremities to such an extent as sometimes to amount to complete paralysis of motion and feeling. Of all the senses the sight suffers most frequently; hearing, taste and smell likewise are often deranged. The manner of the patient is shy and timid—suspicious and morose almost to an insane degree. The sleep is disturbed; headache from slight inconvenient attacks, to the most violent congestive paroxysms, is likely to occur. There is also change of character, from amiable, liberal and ingenious, to peevish discontent and selfish exclusiveness. The most common and terribly harrassing mental affection is melancholy. In fact, hypochondriasis is supposed to be the disease in many instances, and the patient treated for it. We should never forget, however, that hypochondriasis is a *symptom* instead of a disease. The memory and intellect seldom escape long. They are drawn into the general ruin and soon wrecked, so that many of these miserable creatures find their way into the lunatic asylums of the land. The reader of Lallemand will scarcely fail to be forcibly reminded of Bennett. The general effect of seminal disease in men are very similar, according to Lallemand's description, to those so graphically sketched by Bennett as arising from diseases of the uterus in females.

The anatomy of spermatorrhea is inflammation almost if not quite universally. This inflammation commencing in blennorrhagia, or hyper-excitement from any cause, creeps along the mucous membrane of the urethra to the vesiculæ seminales, and into the follicles of the prostate gland, and into the mucous canals of the testicles through the vas deferens, impairing the secretory and excretory capacities of those organs. The semen is increased in quantity, becomes more thin, until it loses all virility, and failing to produce any stimulating effects, is evacuated in the unnatural modes above described.

Frequent causes are gonorrhea, cutaneous eruptions extending to the mucous membrane of the urethra, irritation of the rectum from ascarides, inflammation, stricture, obstinate constipation, hemorrhoids, etc. Masturbation, venereal excesses, lascivious reading and thoughts, various medicines, as cantharides, ergot, nitrate potassa, coffee, tea and many other articles, sebacious collections under the prepuce and glans penis in phymosis, hereditary tendencies, long continued continence, etc., are fruitful operating causes of this terrible affection.

The treatment is divided into general and local. The first has reference to the management of symptoms not immediately connected with the parts most intimately concerned. We should well consider every case in all its bearings, and apply remedies for the promotion of the general health.

The circumstances which attend spermatorrhea as causes and consequences are very various and complicated, as well as important, and will require the skillful application of general principles for their management. But it is to judicious local treatment we must look for the most marked results. Lallemand's mode of cauterizing the urethra is suited to perhaps most cases, and in making this application or resorting to any local means of cure we should remember that we do it for the relief of local inflammation of the urethra and modification of the irritability of the prostatic follicles, the canals of the vesiculæ seminales and perhaps the vas deferens. The same effect may be produced in some instances by introducing the catheter and allowing it to remain in position an hour more or less every day, or every other day, according to the effect it produces. Mr. Solly, of St. Thomas' Hospital, thinks that cauterization will cure almost every case if judiciously applied. We think that in cases to which the nitrate is applicable and would make a cure, it is applied so frequently as to often fail. The effects of each application should entirely subside for some time before it is again used; so long, in fact, as improvement continues. Sometimes the improvement is not apparent for several days after the cauterization. Acupuncture of the prostate in three or four different places from the perineum, with fine needles, allowing them to remain for two or three hours, will often do good when it is very sensitive and irritable. An issue kept open for some time over the same organ also does a great deal of good. Dr. Wilson uses the nitrate of silver in solution, applying through glass or silver canula so as to make a direct application to the part without dilution or chemical alteration. These would take place if it were injected. Local treatment applied to the rectum when it is the seat of stricture, fissure, hemorrhoids, ascarides or other irritation, is indispensably necessary to the successful operation of other means, and will in itself often be sufficient to produce a completely favorable change.

